# Consciousness in Non-Epileptic Attack Disorder

**DOI:** 10.3233/BEN-2011-0315

**Published:** 2011-03-29

**Authors:** M. Reuber, M. Kurthen

**Affiliations:** ^1^Academic Neurology UnitUniversity of SheffieldRoyal Hallamshire HospitalSheffieldUK; ^2^Swiss Epilepsy CentreZürichSwitzerland

**Keywords:** Epilepsy, elderly, age, prevalence, misdiagnosis

## Abstract

Non-epileptic attack disorder (NEAD) is one of the most important differential diagnoses of epilepsy. Impairment of consciousness is the key feature of non-epileptic attacks (NEAs). The first half of this review summarises the clinical research literature featuring observations relating to consciousness in NEAD. The second half places this evidence in the wider context of the recent discourse on consciousness in neuroscience and the philosophy of mind. We argue that studies of consciousness should not only distinguish between the ‘level’ and ‘content’ of consciousness but also between ‘phenomenal consciousness’ (consciousness of states it somehow “feels to be like”) and ‘access consciousness’ (having certain ‘higher’ cognitive processes at one’s disposal). The existing evidence shows that there is a great intra- and interindividual variability of NEA experience. However, in most NEAs phenomenal experience – and, as a precondition for that experience, vigilance or wakefulness – is reduced to a lesser degree than in those epileptic seizures involving impairment of consciousness. In fact, complete loss of “consciousness” is the exception rather than the rule in NEAs. Patients, as well as external observers, may have a tendency to overestimate impairments of consciousness during the seizures.

## 1. Introduction

Non-Epileptic Attack Disorder (NEAD) is one of the most important differential diagnoses of epilepsy. A recent study, which reportedly captured all patients with a blackout first presenting to a neurologist, emergency room or primary care physician, suggested that 57.4% had epilepsy, 22.3% had fainted and 18.0% had had non-epileptic attacks [40].

Non-epileptic attacks (NEAs) superficially resemble epileptic seizures, but are not associated with ictal electrical discharges in the brain [49]. NEAs can be defined positively as episodes of paroxysmal impairment of self-control associated with a range of motor, sensory and mental manifestations, and representing an experiential or behavioral response to distress caused by internal or external stimuli. The overwhelming majority of NEAs are considered manifestations of dissociative or conversion disorders in the current diagnostic manuals [2,80]. Previous review articles have described the diagnostic approach to NEAD [22,58], the clinical picture [56], aetiology [57], and treatment of NEAD [45,46,59]. Here we focus on impairment of consciousness, the most prominent clinical feature of the condition.

The term 'consciousness' is difficult to define because it has different meanings depending on the context in which it is used. The use of 'consciousness' in everyday discourse differs from how the term is applied in psychology, cognitive neuroscience, or the philosophy of mind. In neuroscience, where the term is used much more reluctantly than often assumed, it is generally accepted that consciousness, like other mental phenomena, is dependent on brain functions. Since the scope of neuroscience is limited to insights into the *physical* realm, the more general question of how this 'dependence' is characterised has traditionally been left to the philosophy of mind. Many philosophers favor the weakly materialistic hypothesis that conscious states are supervenient on cerebral states. Supervenience as

ISSN 0953-4180/11/$27.50 © 2011 – IOS Press and the authors. All rights reserved an asymmetrical dependence relation holds that there can be no change in mental (or, specifically, conscious) states unless there is also a change in cerebral states. Neuroscientific research has to confine itself to the identification of the "neural correlate of consciousness" (NCC) [23]. The NCC is defined by mapping states of a neural system to states of consciousness, where the respective state of the neural system is sufficient for the corresponding state of consciousness [21]. Neuropsychological and physiological studies suggest that the NCC is neither a property of the brain as a whole nor a function of a single consciousness 'centre' [24]. Rather, the NCC is established by the interaction and temporal coordination of a wide range of neural subsystems of the human brain (including but not limited to those underpinning sensation, attention, voluntary movement and memory).

But how is 'consciousness' used in clinical neurology? The 1981 classification of the International League Against Epilepsy (ILAE) links consciousness to the patients' degree of awareness and/or responsiveness. While responsiveness is understood as the ability to carry out simple commands or willed movements (intention and perception), awareness is defined as the patients' contact with the event and its recall (memory) [26]. Recently, an alternative model of consciousness has been proposed in epileptology. This model [18] is based on the observation that the level of general awareness and the "subjective contents" of consciousness are supported by different neurobiological systems. Whereas the level of consciousness depends on the functional state of ascending ponto-mesodiencephalic reticular pathways and widespread thalamo-cortical projections, subjective experiences involve the temporo-limbic modulation of a range of cortical activations [16,18,51]. In keeping with this bi-dimensional interpretation, this model distinguishes between 'quantitative' features (the level of consciousness) and 'qualitative' aspects (the content of consciousness). The level of consciousness ranges from alertness to coma, and is reflected by the patient's responsiveness. The content comprises of the patient's subjective experiences during the seizure. The self-report Ictal Consciousness Inventory (ICI) is a quantitative measure based on this model [19].

To date, only a small number of studies have focused directly on the topic of consciousness in NEAD. However, a number of studies have touched on aspects of consciousness in this disorder. The first half of this article will summarise this literature. The second half will place this evidence in the wider context of the recent discourse on consciousness in neuroscience and the philosophy of mind.

## 2. Clinical studies

### 2.1. Ictal consciousness

NEAD is diagnosed most commonly in patients whose attacks involve impairment of consciousness [56]. However, the diagnosis has also been applied to purely motor, sensory or experiential attacks akin to (epileptic) simple partial seizures [49]. Studies using cluster analysis suggest that several types of NEAs can be differentiated on the basis of visible seizure manifestations [3,34]. The commonest semiology involves excessive movement of limbs, trunk and head. Seizures with stiffening and tremor, or seizures with atonia are less frequent in most series [56]. Impairment of consciousness occurs in all three seizure-types.

Whilst patients with NEAD are more likely to stress in interaction with the doctor than patients with epilepsy that they are "completely out" in their seizures and are more likely to deny *any* subjective seizure experience [61,64], a number of studies suggests that ictal impairment of consciousness is typically less profound in NEAD than in (complex partial or generalised) epileptic seizures. Purposeful movements or signs of reactivity may be observed whilst patients appear unconscious [35,50]. Eye opening may be resisted [30, 35,48]. The pupillary light response is preserved [63]. Cyanosis is unusual in NEAs [38]. A study in which 66 patients with epilepsy and 29 patients with NEAD completed one Ictal Consciousness Inventory (ICI) for a total of 167 seizures showed that, at the group level, patients with NEAD reported a higher 'level' of ictal consciousness (ICI-L, $p = 0.01$)) and a greater variety of experiential 'content' (ICI-C, $p = 0.01$). The findings were the same when patients with NEAD were compared with the subgroups with temporal lobe seizures ($n = 52$) or idiopathic generalised epilepsy ($n = 47$). However, there were no significant differences between patients with NEAD and frontal lobe seizures ($n = 14$) [17].

In keeping with these findings based on self-report, a study in which patients with seizure-related impairment of consciousness underwent an ictal assessment procedure showed that 48% of patients with NEAs but only 18% of patients with complex partial seizures were able to follow simple commands [5]. Typically, NEAs impaired recall of ictal experiences to a lesser extent than epileptic seizures. The study cited above showed that 63% of patients who had just experienced a NEA but only 4% of patients who had had a complex partial seizure could recall at least two memory items of a neu-

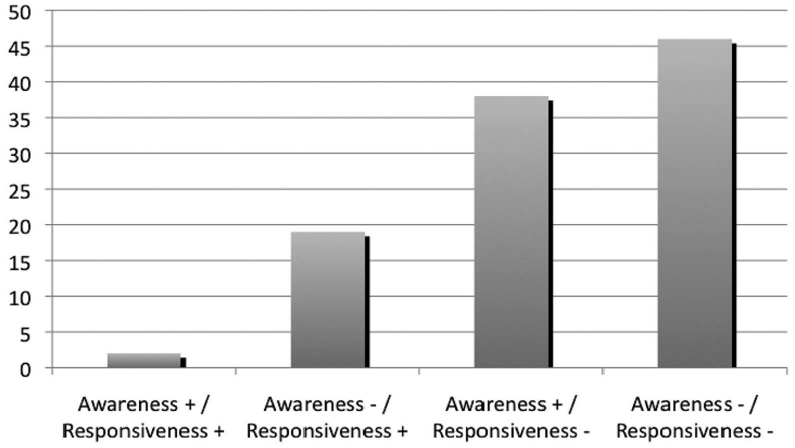

Fig. 1. Percentages of 58 consecutive patients with NEAD self-reporting ‘awareness’ and ‘responsiveness’ during their seizures.

ropsychological testing procedure [5]. Unfortunately, the methodology did not allow the authors of the studies to determine whether the ictal memory problems described by a substantial minority of patients with NEAD and the majority of patients with epilepsy were due to ictal problems with storage of information or to difficulties with post-ictal retrieval. A study in which patients with NEAD or epilepsy were encouraged to recollect details of events which took part during their seizures under hypnosis, suggests that the ictal memory deficits associated with NEAs were at least partly due to the latter: in the NEAD (but not the epilepsy) group, recall for ictal events improved significantly with hypnosis. The hypnotic recall procedure had a sensitivity of 100% and specificity of 85% in the differentiation of NEAD and epileptics seizures [44].

We have carried out two studies based on patients’ self-report of their impairment of consciousness in NEAD. The first was a retrospective analysis of detailed records of seizure descriptions recorded by a single psychotherapist and provided by 58 consecutive patients seen in our psychotherapy program. This study revealed that the largest subgroup of patients said that they were neither ‘aware’ (able to recall all of their seizure experience) nor ‘responsive’ (able to respond at least partially with or without being able to recollect this later) in at least one part of their seizures. However, many other patients reported that they remained at least partially ‘aware’ or ‘responsive’ throughout their seizures. Five per cent had more than one seizure type and were included in different categories (Fig. 1, unpublished data).

The second study was based on the analysis of 100 responses from patients with video-EEG proven NEAD to the 98-item Paroxysmal Event Profile (PEP) questionnaire. The profile asks respondents to rate statements such as “My attacks often come on when I am asleep” on a five-point Likert scale (“always”, “frequently”, “sometimes”, “rarely”, “never”). The PEP questionnaire combines questions from a range of sources. It lists symptoms of panic disorder, derealisation, depersonalisation (PTSD) from the ICD-10, DSM-IV and Present State Examination, the symptoms of dissociation captured by the Dissociative Experience Scale Taxon, DES-T [76,77], questions previously found to distinguish between generalised tonic clonic seizures and syncope [36,66], or thought to differentiate between epilepsy and NEAD [73]. A ‘dissociation’ score was based on the eight items included in the DES-T. An ‘anxiety’ score including physical and mental manifestations and comprised of 14 items. 86 of the patients also provided responses to the 34-item Paroxysmal Event Observer (PEO) questionnaire completed by someone who had witnessed their attacks [62]. Table 1a shows patients’ answers to questions relating to impairment of consciousness. Table 1b shows relevant responses on the PEO questionnaire.

Firstly, the results of this study reflect the heterogeneity of the disorder. Individual patients typically experience a range of seizure severities or symptoms. The “never” and “always” options only made up a minority of responses. Secondly, the findings are consistent with the idea that over one half of NEAs do not involve complete loss of awareness and reactivity. The results of the PEO questionnaire raise the possibility that witnesses underestimate patients’ awareness during their seizures.

Table 1
Respondents replies to some of the items relating to impairment of ictal awareness on the PEP (Table 1a) and PEO questionnaires (Table 1b)

Table 1a

| Item | Always | Frequently | Sometimes | Rarely | Never |
|---|---|---|---|---|---|
| During my attacks I can see or hear people around me | 22% | 17% | 26% | 9% | 26% |
| During my attacks I have no idea what is happening around me | 30% | 25% | 26% | 5% | 14% |
| In my attacks I am conscious but I can't react to things | 28% | 20% | 28% | 1% | 23% |
| In my attacks I drift in and out of consciousness | 28% | 33% | 17% | 4% | 18% |
| I am aware of shaking uncontrollably in the attacks | 16% | 11% | 33% | 4% | 36% |
| Afterwards I have no idea I've had an attack | 11% | 12% | 25% | 18% | 34% |
| After my attack I find myself somewhere and I don't know how I've got there | 8% | 6% | 19% | 10% | 57% |

Table 1b

| Item | Always | Frequently | Sometimes | Rarely | Never |
|---|---|---|---|---|---|
| The attacks involve loss of awareness or the ability to react to things | 63% | 12% | 13% | 1% | 10% |
| The attacks involve a complete loss of consciousness or blackout | 56% | 19% | 8% | 5% | 12% |

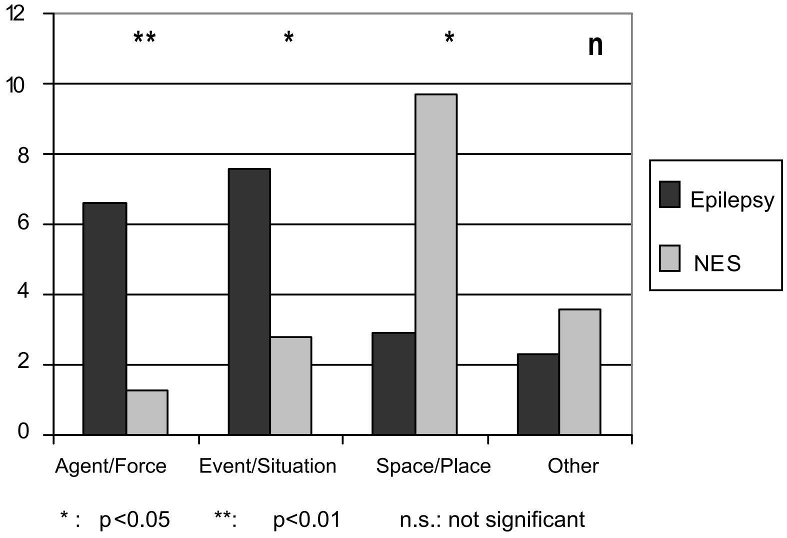

Fig. 2. Preference of metaphoric conceptualisations by patients with epilepsy or NEAD (from [53]).

Our study showed that there was a positive correlation between the summary dissociation and anxiety scores ($r = 0.514$, $p < 0.001$, see Fig. 3) [62].

### 2.2. Subjective seizure experience

Many patients describe physical symptoms of panic or hyperventilation during their seizures, often with few mental symptoms or little cognitive awareness of anxiety [31,70,75,79]. Indeed, NEAs have been interpreted by some as a dissociative response to physical arousal, or as panic attacks without panic [31]. Figure 3 suggests that this interpretation may be appropriate, however, only for one group of patients with NEAD. In other patients impairment of consciousness seems to occur in the absence of symptoms of dissociation or anxiety/arousal. Even in the absence of panic symptoms, most patients experience their seizures as confusing and beyond their control [32,74].

Inspired by a German study [73], we used a cognitive linguistic technique to gain a better understanding what it is like to experience a NEA [53]. Having identified all seizure metaphors used by 21 patients (13 with NEAD, 8 with epilepsy) in transcripts of 30-minute encounters with a neurologist, a linguist blinded to the patients' diagnosis categorised the metaphors into different conceptualisations. 80.8% of 382 metaphors conceptualised seizures as an agent/force, event/situation or space/place (see Table 2). Most patients used metaphors from all categories, but the profile of metaphor choice differed significantly between the epilepsy and NEAD groups (Fig. 2). Patients with epilepsy preferred metaphors depicting the seizure as an agent/force or event /situation. In contrast, patients with NEAD more often used metaphors of space/place. Logistic regression analyses correctly predicted the diagnosis of NEAD or epilepsy in 85.7% of cases.

This observation is in keeping with another study focusing on the use of the medical labels which found that patients with NEAD were more likely than those with epilepsy to resist terms such as “seizure” or “attack” which imply that ictal symptoms are the consequence of an external agent acting on the patients’ body [54]. It also fits with the findings of a study in which 48 patients with video-EEG proven epilepsy or NEAD were contacted after a major earthquake: 23% of patients with epilepsy but none with NEAD volunteered that they thought that they were going into a seizure when they first became aware of the earthquake [78].

### 2.3. *Interictal observations*

A discussion of consciousness in relation to NEAD would not be complete without reference to interictal observations [57]. The curious combination of a history of trauma or conflict and the missing awareness of its pathogenic relevance for apparently physical symptoms is one of the observations which first gave rise to the concept of conversion disorder [28,29].

More recently, several studies have confirmed that many patients with NEAD have experienced significant trauma in their lives [13,27,60]. A sizable subgroup has suffered abuse or neglect in childhood [1,11,65]. The onset of seizures is often precipitated by negative life events [14]. However, whilst patients with NEAD more commonly report negative life events in the year prior to the onset of their seizures [8], they are more likely to deny life stresses and less likely than patients with newly developed epilepsy to attribute their seizures to ‘psychological’ causes [72]. In some cases the missing ‘conscious’ link between a life event or traumatic experience may be related to the fact that the event is related to an ‘unspeakable’ dilemma [33]. The finding that over 90% of patients with NEAD score in the alexithymic range on the Toronto Alexithymia scale offers an alternative explanation for this finding [7]. Adverse early experience may cause deficits of emotion perception and processing which make patients less ‘conscious’ of their emotional state in adult life and therefore less likely to accept that emotional distress could trigger physical symptoms [7,39].

Two experimental studies provide some first insights into the neurobiological underpinnings of these clinical observations. Both suggest that there are abnormalities of interictal preconscious cognitive processing in patients with NEAD (perhaps related to a history of previous trauma). The first measured the eye blink response to startle with or without a weak to moderate stimulus presented briefly before the startle-eliciting stimulus. Patients with NEAD showed impairment of the startle inhibition normally seen after a pre-pulse stimulus [55]. The other study used a masked emotional Stroop test in which colour naming latencies were measured after study participants had been shown neutral, angry or happy faces for 30ms (i.e. not for long enough to enter conscious awareness). Compared to healthy controls, patients with NEAD showed a positive attentional bias to angry faces [4].

## 3. Discussion

### 3.1. *Nosology*

In NEAs, consciousness is thought to be impaired through unknown mechanisms that have been described as processes of dissociation [12]. The DSM-IV defines dissocation as a “disruption of the usually integrated functions of consciousness, memory, identity or perception of the environment” [2]. The concept of dissociation relies on the assumption that the mentioned functions somehow exist as separable modules or entities, which are held together in a well-functioning cognitive system, but which can fall apart under certain pathological conditions. Although the DSM-IV and ICD-10 exclude physical causes before a dissociative disorder can be diagnosed, it is implausible that NEAs are characterized by a *complete absence* of pathological cerebral processes. Quite the contrary: if mental states are asymmetrically dependent on brain states, then some pathological cerebral process must occur, namely there must be a NC of the mental alterations, subjective symptoms and behavioral manifestations that characterise NEAs.

The Standardized Clinical Interview for DSM-IV Dissociative Disorders identifies five different components of dissociative disorders: depersonalization, derealisation, amnesia, identity confusion and identity alteration [71]. The DSM-IV explains that dissociation is not necessarily pathological. In keeping with this notion, the Dissociative Experience Scale (DES), the most widely used measure of dissociation, was conceived as an instrument to capture the full spectrum of dissociative experience ranging from ‘normal’ experiences such

Table 2
Metaphoric conceptualisation of seizures (from [53])

| Category | Seizure as an agent/force | Seizure as an event/situation | Seizure as a space/place | Other |
|---|---|---|---|---|
| Grammatical subject | Seizure | Seizure | Patient | Variable |
| Semantic agency | With the seizure | Variable | With the patient | Variable |
| Examples | seizures come, go, come in, come on, come up, creep up on you, get you, try to do things, set off, are sent in, are straight there, are fought, counteracted, contained, are let pass, wear off | seizures happen, occur, take place, are due, start, finish, go on, carry on, develop, are experienced, witnessed, handled, controlled, stopped, avoided/ put off, are brought on, run their course | drifting off, being off somewhere else, going, going off, being gone, coming back, coming round, coming to, going down, being down, not being there, being out into seizures, in seizures, out of seizures, within seizures, through seizures | seizures are started up, are fixed, like an electrical charge, like the lights are on but nobody's at home, like something going off, like shutting a computer off, like cold or hot water on the top of your head, are as if your head carries on without you |

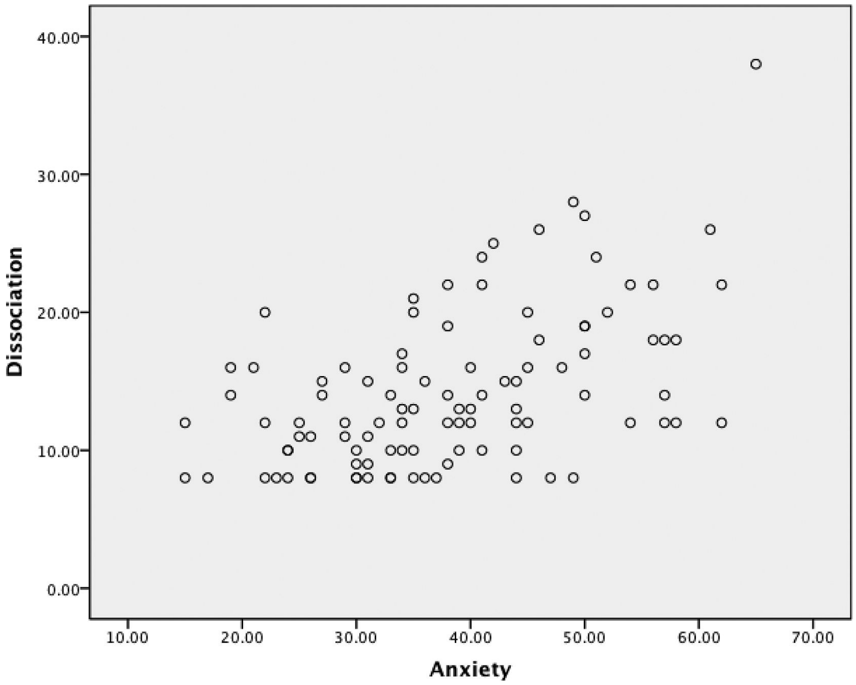

Fig. 3. Positive correlation of the number of ictal symptoms of anxiety and dissociation reported by 100 patients with NEAs ($r = 0.514$, $p < 0.001$).

as absorption at one end to pathological states (such as having multiple identities) on the other [6]. However, subsequent studies in clinical and non-clinical populations have suggested that 'normal' and 'pathological' dissocation may be qualitatively different. The DES-Taxon (based on a subset of eight questions about more abnormal manifestations of depersonalisation, derealisation and amnesia from the DES) has been proposed as a more specific measure of pathological dissociation [77].

More recently, an alternative subdivision of dissociative symptoms has been proposed: based on neurobiological arguments, manifestations of 'detachment' have been separated from those of 'compartmentalisation' [37]. Dissociative 'detachment' has been thought to encompass depersonalization, derealisation and similar phenomena such as out-of-body experiences (i.e. phenomena associated with abnormal distancing of self and environment). The concept of 'detachment' overlaps with manifestations of peri-traumatic dissociation (when individuals dissociate during or immediately after a traumatic event). 'Compartmentalisation' refers to dissociative amnesia and other neurologically unexplained symptoms including paralysis, sensory loss, gait disturbance, aphonia or pseudo-hallucinations (for instance the disintegration functions such as vision/attention/object naming in a patients with dissociative visual loss). In this hypothesised bipartite model of dissociation, NEAD have been attributed to compartmentalisation [37]. Compartmentalisation phenomena are thought to relate to deficits in the integration of neural systems normally amenable to deliberate control [15], whereas detachment experiences are interpreted as a result of the top-down (frontal) inhibition of limbic emotional systems, accompanied by activation of the right prefrontal cortex [67]. A number of studies provide evidence for an inhibitory mechanism of detachment in the context of high emotion and arousal in patients with persistent depersonalisation. A neuroimaging study in which patients were compared with healthy controls showed reduced activation in brain areas associated with emotional experience and increased activation in regions associated with emotional regulation [52]. In a study based on the measurement of skin conductance, patients showed increased response latency to unpleasant stimuli compared to healthy controls and patients with anxiety disorders [68]. There was a steady decline in noradrenaline levels with increasing symptoms of depersonalisation in patients with depersonalisation disorder [69].

The notions that 'dissociation' can be subdivided into 'detachment' and 'compartmentalisation' and that NEAs are associated with the latter have been tested. Patients with NEAD scored more highly on a measure of 'compartmentalisation' than a measure of 'detachment' when compared to epilepsy controls. However, this difference was no longer significant when anxiety and depression were controlled for [47]. The lack of positive findings in this study does not mean that impairment of consciousness in NEAD is not related to the hypothesised processes of dissociation as outlined above. Patients with epilepsy experience dissociative symptoms for different reasons and may not have been a suitable control group.

There are many reasons why this and other studies using self-report tools of dissociation in patients with NEAD have not produced clearer results. These include the difficulties with the concepts of 'normal' and 'pathological' dissocation, 'detachment' and 'compartmentalisation' and the measurement of these experiences. Another important clinical reason is the heterogenity of NEA disorders. The correlation between anxiety symptoms and symptoms of dissociation demonstrated above indicates that there are some NEAD patients with high levels of anxiety and with frequent and numerous dissociative symptoms and others with few or no manifestations of anxiety or dissociation. This raises the possibility that NEA-related impairments of consciousness occur for different reasons.

In some patients NEAs are likely to occur at times of high anxiety. Patients in this subgroup may experience their seizures as a re-traumatisation and may derive symptomatic benefit from ictal detachment underpinned by the inhibition of limbic emotional systems. In these patients postictal amnesia may not (only) be explained by 'compartmentalisation' (inaccessibility of memories which were laid down during the seizure) but by the fact that ictal memories were not secured in the first place or laid down in a fragmented fashion (akin to peritraumatic memories). Other patients with NEAD may have a very low tolerance of emotional fluctuation and may readily 'compartmentalise' without ever becoming aware of anxiety or other unpleasant symptoms of emotional distress.

### 3.2. *Philosophical contributions*

The issue of consciousness in NEAD raises a number of conceptual and methodical problems. As stated above, consciousness is not a single entity, but rather a complex of heterogeneous phenomena like wakefulness, awareness, the capacity for integrated behavior, conscious cognitive capacities like perception, thought, and memory, self-reflection, and others. In the philosophy of mind, it is common to distinguish, in variable terminologies, nonconceptual from conceptual conscious episodes (see [42] for a summary). Best known is Block's (1995) distinction between 'phenomenal' consciousness and 'access' consciousness [9]. A state is nonconceptually, phenomenally conscious if it has "experiential properties", that is, if there is something "it is like to be" in that state *for* the respective organism. It somehow "feels to be" in a certain phenomenally conscious state if, for example, we experience pain or if we sense an object as having a certain colour. A state is access-conscious, on the other hand, insofar as it is relevant for reasoning and/or the control of action of the organism *qua content* of the respective state (Block 1995). Thoughts and beliefs, which are essentially characterised by their content, are paradigmatic for access-consciousness. To be access-conscious largely means to have certain 'higher' cognitive processes at one's disposal, while to be phenomenally conscious means to experience a certain phenomenal quality. This philosophical distinction between conceptual and nonconceptual consciousness differs from the Cavanna and Monaco model of consciousness mentioned above [18]. In that model, the distinction between conceptual and nonconceptual consciousness is largely lost, as the term "subjective content" illustrates, while a "quantitative" aspect of consciousness is introduced in terms of wakefulness, general awareness, or vigilance. These "quantitative" aspects, on the other hand, are somehow implicitly presupposed in Block's model, which does, of course, primarily consider the awake, healthy person, not a person who is asleep, or in a coma. Vigilance and awareness are aspects of consciousness that belong to a more basic level of functioning, at least when phenomenal consciousness is concerned: we can assume that to have phenomenally conscious states, a certain level of awareness and wakefulness is required as a necessary (but not sufficient) precondition. For access-consciousness, the question of how much 'quantitative' consciousness is required to operate on an access-conscious level is an intricate issue, since at least a major part of complex, integrated behavior can be performed unconsciously. On the other hand, we would probably not be tempted to speak of rational action or meaningful speech (except in a psychoanalytical sense!) in a person who is not awake. Anyway – for a discussion of consciousness in NEAs, it would be desirable to differentiate the quantitative aspects of consciousness from the features of phenomenal and/or access-consciousness, as far as possible. Whereas Cavanna and Monaco apply the terminology of the 'subjective' and the 'qualitative' also to features of access-consciousness, for the philosopher, these terms remain reserved for the characterization of phenomenal consciousness.

### *3.3. Assessment of consciousness*

Aside of the problem of the *definition* of consciousness, there is the issue of how to *empirically assess* conscious phenomena, or how to empirically verify or prove the presence of consciousness. The immediate and direct detection of conscious phenomena is neither possible in clinical settings nor in neuroscientific experiments, because these phenomena are always and only accessed via intermediate overt behavioral or physiological markers: verbal reports, button presses, event-related potentials, EEG patterns, BOLD responses, and the like. Due to the essentially private and/or subjective character of conscious states, it seems methodologically impossible to detect or observe conscious states of other beings directly from a third-person perspective. Actual neuroscientific approaches to consciousness never rest upon direct correlations between neuronal and conscious states, because the reference to conscious states is always mediated by the overt markers listed above. The degree of external inaccessibility seems to be higher for the phenomenal features of conscious states than for access-conscious states or basic 'quantitative' features like vigilance, because the latter, but not the former can be operationalised as content-sensitive or stimulus-related cognitive operations that are, at least in principle, objectively accessible with respect to their non-phenomenal features. In the philosophy of mind, the fundamental inaccessibility of another person's conscious experience has raised the 'other minds problem', namely the question of how one could ever be sure that other people are conscious beings like oneself (see [43] for an application of this question to neurology).

There may be good reasons to prefer an account of behavioral or physiological markers of consciousness to an approach to *consciousness* as such in cognitive neuroscience. As stated above, conscious states can only be accessible by means of their overt 'markers', their behavioral or physiological correlates. This makes conscious states as such methodologically unattractive for cognitive science, to say the least. And there is yet another obstacle. In the interpretation of neuroscientific data – as well as in everyday communication – the problem of authenticity arises: how can we be sure that a person reports the conscious states s/he actually 'had'? Even if the report is sincere, s/he might simply fail correctly to remember the previous conscious states (as is the case in epileptic seizures, where the initial occurrence of an aura may no longer be remembered after secondary generalization of the seizure). Furthermore, there is a more fundamental crux to this issue: could it be that a person is literally mistaken about her own conscious states, even if memory is intact? This is the notorious philosophical issue of the 'epistemic authority' concerning conscious states, a question, which acquires particular significance in contexts where interplay between consciousness and the unconscious is assumed – notably in psychoanalysis, the theory which provided the first serious attempt to explain psychogenic alterations of consciousness. Confronting the inaccessibility of conscious states in cognitive neuroscience and philosophy, even some philosophers have proposed to handle this problem pragmatically by simply taking verbal reports for granted and "accept what people say about their experiences" [10, p. 459]. In that case, however, it has to be borne in mind that explanations based on such evidence can account for consciousness only as far as it is reportable at all, so that it would be more correct to take the authentic verbal report as evidence for the "reportability" of a mental state, not as evidence for its conscious character per se [20].

Hence, if we use overt markers of conscious states with phenomenal features, we have to be aware that the method of assessment constrains the features of consciousness that actually become accessible. For example, in postictal interviews we do not assess ictal consciousness as such, but memory and reportability. Due to these constraints, it is notoriously difficult to disentangle a patient's impairment in cognitive access to or retrieval of previous ictal conscious states from genuine impairments of ictal consciousness. Although in principle, this problem holds both for patients with epilepsy and patients with NEAD, there seem to be interesting differences between these two groups. In relation to epileptic seizures, it seems that a lack of concordance between actual ictal signs of consciousness and postictal reports is mainly due to mnestic impairment. If a patient verbally reports their ongoing aura at the beginning of a secondarily generalised seizure, and then denies the occurrence of that aura in the post-ictal interview, then it seems obvious that the propagated ongoing seizure activity has somehow prevented the formation of a stable memory "trace" of the previous aura experience. We would not assume that the patient erroneously reported his aura, or that he was 'unconscious' while reporting his aura. In NEAs, on the other hand, the pathological process of hypersynchronization is lacking, so that we will have to recourse to other mechanisms to explain why a patient with NEAD who displays ictal conscious behavior will describe themselves as having 'lost consciousness' when interviewed after the seizure. If it is not a mnestic problem, could it be that the patient with NEAD, in contrast to the patient with epilepsy, is literally mistaken about his/her own ictal conscious states? An answer to this question cannot be provided by clinical observation or neuroscientific evidence alone. Our answer to this question will at least partially be determined by our basic everyday intuitions or, if we are theoretically educated, our philosophically substantiated beliefs concerning consciousness and the mind. Just very shortly: to answer this question, we would have to decide not only whether epistemic authority concerning conscious features of mental states is necessarily to be ascribed to the subject of these states, but also whether it makes sense at all to supplement a *real* phenomenal state of a person's mind by an *even more real* phenomenal state that is somehow inaccessible to the person. Dennett, who ridiculed the notion of a 'real' phenomenality over and above the manifestations of mental events in actions and behaviour altogether, took for granted that the idea of an "objectively subjective", that is, of a way things "actually, objectively seem to you even if they don't seem to seem that way to you", would be "bizarre" [25, p. 132]. We will not try to solve this issue in the present paper (see [41] as an attempt), but rather point to the peculiar fact that according to a modern reading of psychoanalysis [81]), the "objectively subjective" ridiculed by Dennett is exactly what is meant by 'the unconscious' in the psychoanalytical sense, namely the unconscious, inaccessible psychic phenomenon that is the basic characteristic of human subjectivity. The very same "objectively subjective" that strikes the analytical philosopher as "bizarre" figures as the basic, unquestionable constellation of the mind for the psychoanalyst.

## 4. Conclusion

What do we make of the findings of the empirical evidence relating to NEAD in the light of these conceptual considerations? Phenomenal consciousness is extremely difficult to assess. If we take for granted what people say about their ictal experiences in NEAs, it seems that typically, phenomenal experience – and, as a precondition for that experience, vigilance or wakefulness – is reduced to a lesser degree in NEAs than in epileptic seizures involving impairment of consciousness. As the study of preferred metaphoric conceptualisations for seizure experiences indicates, there may also be qualitative differences in terms of what "it feels like to be" in a NEA rather than an epileptic seizure, but of course, the question of a subsequent contamination of these experiences by postictal mechanisms of selective retrieval and interpretation always remains open. Concerning the phenomenal ictal features of consciousness, however, 'false negatives' concerning phenomenal consciousness cannot be excluded on the basis of the available evidence: patients may have ictal consciousness, but may be unable to recollect their experience afterwards, or they may even be mistaken about their ictal "objectively subjective" experience. Further studies that would have to compare ictal signs of consciousness and postictal reports on an individual level both in NEAs and epileptic seizures might be able to shed some light on these issues.

As for quantitative features of consciousness and higher, action-related cognitive operations (access-consciousness), we propose to take basic motor and vegetative responses (motor reaction to sensory stimuli, pupillary reactions, and the like) as behavioral or at least overt markers of quantitative consciousness, while more complex behavioral phenomena like adequate responses to linguistic stimuli, spontaneous purposeful movements, or even reasonable speech production might count as tokens of access-conscious behavior. Although the empirical evidence is still rather weak, it seems that there is a wide range of degrees of impairment of the different aspects of consciousness. Initial emphatic reports of "complete" loss of consciousness by patients in clinical encounters with neurologists are likely to be an interactional phenomenon [64]. They often give way to more detailed accounts including recollections of what "it felt like to be" in a NEA with careful and more persistent questioning, or can be overcome with hypnosis if desired. Taken together, the studies discussed above suggest that complete loss of "consciousness" is the exception rather than the rule in NEAs. Patients, as well as external observers, may have a tendency to underestimate the degree of preservation of consciousness during the seizures. The considerable inter- and intra-individual heterogeneity of NEA experiences call for a more nuanced differentiation and nosology of NEAs.

## Acknowledgements

We thank Stephanie Howlett for giving us access to unpublished data and Andrea Cavanna for sharing a manuscript in preparation with us.
